# Probiotic and commensal gut microbial therapies in multiple sclerosis and its animal models: a comprehensive review

**DOI:** 10.1080/19490976.2021.1943289

**Published:** 2021-07-15

**Authors:** Lorrie L. Blais, Theresa L. Montgomery, Eyal Amiel, Paula B. Deming, Dimitry N. Krementsov

**Affiliations:** Department of Biomedical and Health Sciences, University of Vermont, Burlington, VT, USA

**Keywords:** Multiple sclerosis, probiotics, commensals, microbiome, microbiota–gut–brain axis, autoimmune disease, comprehensive literature review

## Abstract

The need for alternative treatments for multiple sclerosis (MS) has triggered copious amounts of research into microbial therapies focused on manipulating the microbiota–gut–brain axis. This comprehensive review was intended to present and systematically evaluate the current clinical and preclinical evidence for various probiotic and commensal gut microbial therapies as treatments for MS, using the Bradford Hill criteria (BHC) as a multi-parameter assessment rubric. Literature searches were performed to identify a total of 37 relevant studies (6 human, 31 animal), including 28 probiotic therapy and 9 commensal therapy studies. In addition to presenting qualitative summaries of these findings, therapeutic evidence for each bacterial formulation was assessed using the BHC to generate summative scores. These scores, which encompassed study quality, replication, and other considerations, were used to rank the most promising therapies and highlight deficiencies. Several therapeutic formulations, including VSL#3, *Lactobacillus paracasei, Bifidobacterium animalis, E. coli* Nissle 1917, and *Prevotella histicola*, emerged as the most promising. In contrast, a number of other therapies were hindered by limited evidence of replicable findings and other criteria, which need to be addressed by future studies in order to harness gut microbial therapies to ultimately provide cheaper, safer, and more durable treatments for MS.

## Introduction

### Multiple sclerosis

Multiple sclerosis (MS) is a chronic autoimmune disease of the central nervous system (CNS) characterized by neuroinflammation, myelin sheath degeneration, axonal loss, and blood–brain barrier (BBB) deterioration.^1^^,2^ Globally, about 2.8 million people are estimated to live with MS.^3^ The disease typically follows four different clinical courses: clinically isolated syndrome (CIS), relapsing-remitting MS (RRMS), primary progressive MS (PPMS), and secondary progressive MS (SPMS), with progressive forms being most severe and refractory to treatment.^1^ As MS progresses, most patients are challenged with chronic pain and fatigue, gradual sensorimotor impairments, bowel and bladder dysfunction, cognitive changes, and overall diminished quality of life.^1,2^

There are currently 15 FDA-approved disease-modifying therapies (DMTs) used to decrease the severity and frequency of MS relapses.^4–6^ These DMTs are generally effective at mitigating MS pathology by suppressing various aspects of the immune system, but they are also expensive,^2^ often accompanied by an array of side effects,^5^ and demonstrate decreased efficacy over time.^6–9^ As such, improved, alternative MS treatments are warranted.

Like most chronic diseases, susceptibility to MS is driven by both genetic and environmental components, with the latter including well-documented risk factors like Epstein–Barr virus infection, vitamin D-insufficiency, and smoking.^10,11^ An emerging putative pseudo-environmental risk factor for MS and other chronic diseases is an imbalance (dysbiosis) in the gut microbiome, a complex ecosystem of trillions of microorganisms inhabiting our intestinal tracts. A generalized mechanism proposed is the bidirectional communication between the CNS and gut by way of the so-called microbiota-gut-brain axis (MGBA).^1,12–14^ Dysbiosis within the gut can promote effector T cell phenotypes toward proinflammatory pathways that subsequently increase intestinal barrier permeability.^15–20^ This enables the release of microbial antigens and intestinal immune cells into circulation, further promoting systemic, low-level inflammation which may contribute to the weakened BBB tight junctions and enhanced T cell autoreactivity observed in MS.^15,16,18–20^ The directionality of whether MS contributes to, as opposed to results from, this dysbiosis, however, is still unclear. Nevertheless, multiple studies^16,18–20^ have characterized MS gut microbiomes as distinct from their healthy control-counterparts, generally possessing an elevated relative abundance of microrganisms associated with inflammation that when transplanted into mice have been shown to exacerbate an experimental animal model of MS, experimental autoimmune encephalomyelitis (EAE).^19^

### Commensal and probiotic gut bacterial therapies

Given the putative role of gut dysbiosis in promoting MS susceptibility, an attractive therapeutic approach would be to restore balance of the microbiome, and/or to take advantage of the intimate cross-talk between the immune system and gut microorganisms to inhibit or skew autoimmune responses.^21–24^ Hence, gut targeted microbial therapies have been gaining traction as alternative or supplemental treatment options for a variety of conditions, including MS.

Gut bacterial commensals are generally beneficial organisms naturally comprising the gut microbiome to maintain a healthy host environment.^22^ Whereas probiotics are defined by the International Scientific Association for Probiotics and Prebiotics (ISAPP) as “live microorganisms that, when administered in adequate amounts, confer a health benefit on the host.”^25^ For the purposes of this review, these are defined as live bacteria that can be supplemented into the microbiome to elicit beneficial changes in the commensal microbial community structure, and/or exert direct beneficial effects on the host.^22,26,27^ It should be noted, however, that the exact distinction between gut bacterial commensals and bacterial probiotics remains arbitrary and far from uniform across studies, organizations, and regulatory guidelines. Nevertheless, both commensals and probiotics have important roles in digestive and immune health, including nutrient and vitamin synthesis, metabolism of host dietary products, intestinal barrier reinforcement, prevention of pathogenic microbe colonization, and anti-inflammatory immunoregulation.^21,22,26,27^Consequently, both have the therapeutic potential to mitigate MS pathology through modulation of the MGBA. This, in combination with accessibility and relatively low costs, makes probiotic and commensal therapies attractive alternative MS treatment candidates.

### Alternative MS therapies: where do we stand?

A number of recent reviews have attempted to compile the growing body of evidence for gut microbiome-targeted therapies, though most of these are far from comprehensive. ^12,26–34^ To date, there have been two published reviews that evaluate the clinical utility of probiotics and explore possible underlying mechanisms, including one systematic review of two clinical and five preclinical studies,^31^ and one recent study with a meta-analysis of three clinical studies and systematic review of 22 preclinical studies.^33^ Though valuable for highlighting probiotic therapeutic efficacy, both of these reviews are exclusively focused on probiotics without consideration of commensal therapy, and neither ranked the current evidence of each specific gut bacterial formulations using a quantitative objective rubric. Addressing the latter is particularly important, given that it helps to identify stronger and weaker areas within the field, highlight discrepancies, and ultimately provide direction for future research.

Accordingly, in this comprehensive review, we attempt to (1) compile the current clinical and preclinical evidence of MS mitigation by probiotic and commensal therapies; and (2) systematically rank the evidence of each gut-bacterial formulation using the Bradford Hill criteria (see below). In doing so, we aim to identify the most promising emerging therapies, as well as to highlight existing shortcomings in the field and emphasize specific foci for future studies.

## Methods

This comprehensive review was originally intended as a systematic review, and therefore registered in the International Prospective Register of Systematic Reviews (PROSPERO; ID# CRD42020206819) following the initial search, but prior to screening articles.

### Search strategies

Searches were conducted by two authors (LB & TM) on August 27, 2020 and January 4, 2021 using four databases: OvidMEDLINE, CINAHL, PubMed, and Web of Science. One paper was identified separately by one author (DK) outside of the search strategy.^35^ Search strategies were tailored to each database using keywords, MeSH and MH headings, truncation, and an English Language filter (Supp. File 1A).

### Selection criteria

Studies from all years were included in this review if they (1) were written or available in English, (2) investigated the effects of probiotic and/or commensal therapy on MS or an MS animal model severity and progression, and (3) utilized an experimental/intervention-based study design. Studies were excluded if they did not meet the inclusion criteria and/or used a non-intervention/experimental study design, including cohorts, cross-sectional studies, case–control studies, case series, and case reports.

### Operational definitions

As mentioned above, the distinction between bacterial probiotics and commensals is not well defined, particularly as it applies to MS. For the purposes of this review, a bacterial therapeutic was considered “probiotic” when meeting evidence level 1–2 based on World Gastroenterology Organization guidelines from the Oxford Center for Evidence-Based Medicine, and “commensal” if falling at evidence level 3 and below where RCTs are lacking.^36^ Study interventions were therefore classified as “probiotic therapy” if researchers supplemented with the following putative probiotics: *Lactobacillus* spp., *Bifidobacterium* spp., *Escherichia coli* Nissle 1917 (*E. coli Nissle 1917), Enterococcus faecium* (*E. faecium*), or *Streptococcus thermophilus* (*S. thermopohilis*); or “commensal therapy” if researchers supplemented with any other species of commensal bacteria, including *Prevotella* spp., *Akkermansia* spp., *Pediococcus acidilactici* (*P. acidilactici), Clostridium butyricum* (*C. butyricum*), and *Bacteroides fragilis* (*B. fragilis*).

There were two animal studies that were exceptions to these classifications, for the following reasons. Both studies introduced putative probiotic *Lactobacillus* spp. via stable colonization by a single inoculation rather than continuous treatment, which is more representative of commensal therapy than a probiotic therapy.^37,38^Additionally, the bacterial strains used in these two studies are not strains recognized as probiotics, but are instead isolates from commensal murine gut microbiota. These two studies were hence classified as commensal therapy.

### Data extraction

Following screening studies for relevance against the selection criteria (LB, TM, & DK), data were extracted from the included studies by two authors (LB & TM) (Supp. File 1). The study metrics extracted included first author, year of publication, DOI, location of study, study design, sample, intervention, duration of study, MS model (for animal studies), measurements/outcomes, statistical methods, and power (for human studies). The study measurement/outcomes extracted included clinical parameters of MS/EAE severity and progression, immune and metabolic indices, microbiome and metabolome parameters, and mechanistic or correlative findings.

### Evaluating quality and evidence of included studies

Included studies were subject to quality and risk of bias (ROB) assessments using the Cochrane ROB tool^39^ for human studies and SYRCLE tool^40^ for animal studies. High quality was assigned to studies with a low ROB, including randomized controlled trials (RCTs) and animal studies that explicitly stated using randomization and blinding measures. Medium quality was assigned to studies with an uncertain ROB, including non-RCT human studies and animal studies that did not explicitly state using randomization and/or blinding measures. Low quality was assigned to studies with a high ROB, including studies with considerable confounding, in addition to not explicitly stating the use of randomization or blinding. These quality assessments were factored into the summative evaluation of each bacterial therapy; therefore, no studies were excluded from analysis on the basis of ROB.

The overall quality and strength of therapeutic evidence provided by each bacterial formulation was assessed using the Bradford Hill criteria (BHC), which includes the following: temporal relationship, strength of relationship, dose–response relationship, replication of findings, biological plausibility, cessation of exposure, specificity of association, and coherence between multiple approaches.^41,42^ The descriptions and numerical designations of each BHC can be found in Table 1. Sufficient evidence (Yes or No) was determined for each criterion (except for replication, see below) and assigned a score of 1 for yes, followed by summation across all criteria to yield a final “BH score” for each therapy. Replication of findings was the most heavily weighted criterion, and was scored as follows: 3 = replicated in human and animal studies, 2 = replicated by different groups, 1 = replicated by the same group, 0 = not replicated, −2 = conflicting findings (not considering lack of effect as conflicting with positive). The calculations are detailed in Supplemental File 2 and summarized in Table 5.

**Table 1.** Bradford Hill criteria numerical designations and descriptions

BHC	BHC #	Description
Temporal relationship	1	*Does the exposure precede the outcome?*
Strength of relationship	2	*Level of evidence, ROB, study quality*
Dose-response relationship	3	*U-shaped, inverse U-shaped, etc.*
Replication of findings	4	*Across studies, across research groups, etc.*
Biological plausibility	5	*Do the findings make biological sense?*
Cessation of exposure	6	*Do the effects change after discontinuation?*
Specificity of association	7	*Does the exposure lead to the outcome?*
Coherence between multiple approaches	8	*How well do different lines of experimentation or observation support one another?*

*BHC*, Bradford Hill criteria; *ROB*, risk of bias.

**Table 2.** Human studies of probiotic therapies: summary of study characteristics and major findings

Study	ROB	Sample	MS Type	Intervention	Timeline	Duration	Major Findings
Kouchaki et al.^60^	Low	18–55 yo *n* = 30/g	RRMS EDSS ≤ 4.5	**T**: Probiotic capsule, 2 × 10^9^ CFU (*L. acidophilus, L. casei, B. bifidum, L. fermentum)* **C**: starch capsule	Thera	Biweekly for 12 wks	*CD*: ↓ EDSS, BDI, GHQ-28, DASS *IM*: ↓ hs-CRP, MDA; ↑ plasma NO *MM*: NA *MC*: NA
Rahimlou et al.^63^	Low	18–50 yo *n* = 35/g	RRMS EDSS ≤ 4.5	**T**: “Protexin”^a^ capsule, 2 × 10^9^ CFU **C**: maltodextrin capsule	Thera	Daily for 6 mos	*CD*: – EDSS; ↓ BDI, GHQ-28, FSS & MPQ *IM*: ↓ IL-6; ↑ BDNF; – NGF *MM*: NA *MC*: NA
Salami et al.^64^	Low	20–60 yo *n* = 24/g	RRMS EDSS ≤ 4.5	**T**: Probiotic capsule, 2 × 10^9^ CFU (*L. plantarum, L. casei, L. reuteri, L. fermentum, B. lactis, B. infantis*) **C**: maltodextrin capsule	Thera	16 wks (freq. not specified)	*CD*: ↓ EDSS, DASS; – BDI, GHQ-28 *IM*: ↓ IL-6, TNF-α, hs-CRP, MDA, 8-OHdG; ↑ IL-10, TAC, GSH, NO *MM*: NA *MC*: NA
Tamtaji et al.^61^	Low	18–55 yo *n* = 20/g	RRMS EDSS ≤ 4.5	**T**: Probiotic capsule, 2 × 10^9^ CFU (*L. acidophilus, L. casei, L. fermentum, B. bifidum*) **C**: starch capsule	Thera	Daily for 12 wks	*CD: –* BMI; no relapses *IM*: ↓ IL-8, TNF-α; – IL-1, PPAR-γ, LDLR *MM*: NA *MC*: NA
Tankou et al.^46^	High	*n* = 7 MS, taking GA *n* = 2 MS, untreated *n* = 13 HC	RRMS	**T**: VSL3,^b^ 3.6 × 10^12^ CFU/d **HC**: VSL3,^b^ 3.6 × 10^12^ CFU/d	Thera	Daily for 2 mos	*CD*: NA *IM*: ↑ freq. IL-10+ Tregs; ↓ freq. intermediate & inflammatory monocytes, costimulatory marker CD80 on classical monocytes, MFI of HLA-DR on myeloid-derived DCs, rel freq. of T_H_1 & T_H_17 cells; ↓ freq. IL-10+ Tregs & ↑ freq. of inflammatory monocytes following cessation; – freq. B cells, NK cells, myeloid or plasmacytoid DCs, naïve CD4 or CD8 T cells, central memory CD4 or CD8 T cells, effector memory CD4 T cells *MM*: ↑ rel. abund. of *Lactobacillus, Streptococcus*, & *Bifidobacterium*, which returned to baseline following cessation *MC*: NA
Tankou et al.^47^	High	*n* = 7 MS, taking GA *n* = 2 MS, untreated *n* = 13 HC	RRMS EDSS = 1.4 ± 0.9	**T**: LBS,^c^ 3.6 × 10^12^ CFU/d **HC**: LBS,^c^ 3.6 × 10^12^ CFU/d	Thera	Daily for 2 mos	*CD*: NA *IM*: ↑ freq. IL-10+ Tregs; ↓ freq. intermediate & inflammatory monocytes, costimulatory marker CD80 on classical monocytes, MFI of HLA-DR on myeloid-derived DCs, rel freq. of T_H_1 & T_H_17 cells; ↓ freq. IL-10+ Tregs & ↑ freq. of inflammatory monocytes following cessation; – freq. B cells, NK cells, myeloid or plasmacytoid DCs, naïve CD4 or CD8 T cells, central memory CD4 or CD8 T cells, effector memory CD4 T cells *MM*: ↑ rel. abund. of *Lactobacillus, Streptococcus*, & *Bifidobacterium;* ↑ rel. abund. of *Veillonellaceae* family & *Collinsela* genus; ↓ rel. abund. of *Akkermansia, Blautia, Dorea, B. adolescentis*; microbiome changes returned to baseline following cessation & were associated with changes in stool metabolic profile *MC*: ↓ expression of MS risk allele HLA.DQA.1, & *HLA.DPA1, ILGST, MALT1, LGALS3* in monocytes; ↑ expression of *IL-10RA, LILRB2, CYBB*; most returned to baseline following cessation.

All findings are reported with respect to control group(s) unless otherwise indicated.

**^a^Protexin **= *B. subtilis* PXN 21, *B. bifidum* PXN 23, *B. breve* PXN 25, *B. infantis* PXN 27, *B. longum* PXN 30, *L. acidophilus* PXN 35, *L. rhamnosus* PXN 54, *L. helveticus* PXN 45, *L. salivarius* PXN 57, *L. lactis* ssp. lactis PXN 63, *S. thermophilus* PXN 66, *L. casei* PXN 37, *L. delbrueckii* ssp. bulgaricus PXN 39, *L. plantarum* PXN 47.

**^b^VSL3 **= *L. paracasei* DSM 24732, *L. plantarum* DSM 24730, *L. acidophilus* DSM 24734, *L. delbruckeii* subsp. *bulgaricus* DSM 24734, *B. longum* DSM 24736, *B. infantis* DSM 24737, *B. breve* DSM 24732, *S. thermophilus* DSM 24731.

**^c^LBS **= see ^b^VSL3.

**Key**: ↓ decreased; ↑ increased; – no change or no difference compared to control; NA, not applicable to this study.

**Abbreviations**: ROB, risk of bias; MS, multiple sclerosis; yo, y old; g, group; RRMS, relapsing-remitting multiple sclerosis; GA, glatiramer acetate; HC, healthy controls; EDSS, Expanded Disability Status Scale; T, treatment group; C, control; CFU, colony forming units; Thera, therapeutic; wks, weeks; mos, months; freq., frequency; CD, clinical disease; IM, immune/metabolic; MM, microbiome/metabolome; MC, mechanistic/correlative; BDI, Beck Depression Inventory; GHQ-28, General Health Questionnaire-28; DASS, Depression Anxiety Stress Scales; hs-CRP, high-sensitivity C-reactive protein; MDA, malondialdehyde; NO, nitric oxide; FSS, Fatigue Severity Scale; MPQ, McGill Pain Questionnaire; IL, interleukin; BDNF, brain-derived neurotrophic factor; NGF, nerve growth factor; TNF-α, tumor necrosis factor alpha; 8-OHdG, 8-Oxo-2ʹ-deoxyguanosine; TAC, total antioxidant capacity; GSH, glutathione; BMI, body mass index; HLA-DR, human leukocyte antigen-antigen D related; DC, dendritic cell; rel. abund., relative abundance; NK, natural killer.

**Table 3.** Animal studies of probiotic therapies: summary of study characteristics and major findings

Study	ROB	Sample	MS Model	Intervention	Timeline	Duration	Major Findings
Maassen et al.^59^	UN	F SJL mice 8–12 wko *n* = 6/g	PLP-induced EAE	**T1**: *L. reuteri* ML1 **T2**: *L. casei* 393 **T3**: *L. plantarum* NCIB 8826 **T4**: *L. murinus* CNRZ Each 10^10^ CFU o.g. **C**: NaCO_3_	Proph	Every other day for 5 admins	*CD*: ↑ CDB (T1), weak ↓ (T2), ↓ (T4), – (T3) *IM*: NA *MM*: NA *MC*: NA
Salehipour et al.^50^	Low	F C57BL6 mice 8–10 wko *n* = 8/g	MOG-induced EAE	**T1**: *L. plantarum* A7, 10^9^ CFU o.g. **T2**: *B. animalis* PTCC 1631,10^9^ CFU o.g. **T3**: T1+ T2 **C**: sterile saline	Thera	Daily for 22 d	*CD*: delayed EAE onset (T1-T3), T3 more pronounced; ↓ EAE CS, CDI, incidence, infiltration of MNCs, and demyelination (T1-T3), T3 more pronounced *IM*: ↓ ASP, IFN-γ, IL-6, IL-17 (T1-T3), T3 more pronounced; ↑ % CD4+ CD25+ Foxp3+ Tregs, IL-4, IL-10, TGF-β (T1-T3), T3 more pronounced *MM*: NA *MC*: ↑ expression of *GATA3, FoxP3* and ↓ expression of *Tbet, ROR*γt (T1-T3), T3 more pronounced
He et al.^75^	UN	F C57BL6 mice 10–12 wko *n* = 37–40/g	MOG-induced EAE	**T**: *L. reuteri DSM 17938* 100 µL of 10^8^ CFU o.g. **C1**: 100 µL MRS media + EAE, o.g. **C2**: normal control	Thera	Daily for 20 d	*CD*: ↓ CDS, EAE incidence, maximum CS, inflammatory cell infiltration *IM*: ↓ CD3 + T cells & CD68+ macrophages in spinal cord, % and abs. # of T_H_1 and T_H_17 cells, IL-17, IFN-γ, ↓ % MOG_35-55_-specific splenocytes *MM*: reversed EAE rel. abund. changes (↑ Bacteriodetes, Proteobacteria, Deferribacteres); *Bifidobacterium, Lactobacillus, Prevotella*, & S24-7 = negative correlation w/ CS *MC*: NA
Goudarzvand et al.^52^	UN	M Wistar rats 8–10 wko *n* = 8/g	GID	**T1**: *L. plantarum* **T2**: *Bifidobacterium* B94 Each 1.5 × 10^8^ CFU/mL orally **C1**: GID only **C2**: sterile saline, no GID	Thera	Daily for 28 d	*CD*: – traveled distance, escape latency, or swimming speed *IM*: NA *MM*: NA *MC*: NA
Consonni et al.^53^	Low	F Lewis rats 6–8 wko *n* = 6–9/g (**T1-T4**), *n* = 18/g (**T5-T6**)	gpMBP-induced EAE	**T1**: *L. crispatus* LMG *P*-23257 **T2**: *L. rhamnosus* ATCC 53103 **T3**: *B. animalis* subsp. lactis BB12 **T4**: *B. animalis* subsp. LMG S-28195 **T5**: T1+ T2 **T6**: T3+ T4 Each 2 × 10^9^ CFU/300 µL orally **C**: vehicle	Proph & Thera	15 doses over 3 wks (5 doses pre-EAE)	*CD*: ↓ EAE incidence & median score at peak (T1-T6); ↓ myelin loss, astrocytosis, & spinal cord immune cell infiltration (T5/T6); delayed EAE onset (T1,T2, T5, T6); dose-response relationship observed with T5/T6 *IM*: ↓ proliferative response to MBP, IFN-γ, TNF-α, IL-17 (T5/T6); ↑ TGF-β, IL-16 (T5/T6) *MM*: NA *MC*: NA
Baken et al.^73^	UN	M Lewis rats 6–8 wko *n* = 8/g	gpSCH-induced EAE	**T**: *L. casei* strain Shirota, 1 mL of 1 × 10^9^ CFU/mL **C**: 1 mL saline/peptone	Proph & Thera	Daily for 35 d (starting 8 d pre-EAE)	*CD*: ↓ body weight; ↑ EAE incidence, duration, CDS, & CDI; earlier EAE onset *IM*: NA *MM*: NA *MC*: NA
Gharehkhani Digehsara et al.^71^	UN	F C57BL6 mice 8–10 wko	Cuprizone (4 wks)	**T1**: *L. casei* 4 wks, cuprizone 4 wks **T2**: Cuprizone 4 wks, *L. casei* 4 wks **T3**: Cuprizone 4 wks, *L. casei* 4 wks w/ Vitamin D3 (20 IU/d) **C1**: *L. casei* 4 wks, 1 × 10^9^ CFU/mL **C2**: Cuprizone 4 wks, 0.2% w/w All admin orally **C3**: normal control	Proph & Thera		*CD*: more normal & significant Y-maze alternation behavior (T1-T3) *IM*: ↓ IL-17 (T1-T3), T3 more pronounced; ↑ TGF-β (T1-T3) *MM*: NA *MC*: ↓ expression of *IDO* gene & miR-155 (T1-T3, C2); ↑ expression of miR-25 (C2), trending in T1-T3
Kobayashi et al.^68^	UN	M & F Lewis rats 7 wko & 2 wko *n* = 8/g	gpSCH- or gpMBP-induced EAE	**T1**: *L. casei* strain Shirota in M rats (7 wko), 1–2 × 10^9^ CFU o.g. **T2**: *L. casei* strain Shirota in F rats (7 wko), 1–2 × 10^9^ CFU o.g. **T3**: *L. casei* strain Shirota in M & F rats (2 wko), 9.2–10.1 × 10^9^ CFU o.g. **T4**: *B. breve* strain Yakult in M & F rats (2 wko), 5.0–6.9 × 10^9^ CFU o.g. **C**: 0.5 mL saline/peptone	Proph & Thera	Daily T1-T2: 35 d (starting 7 d pre-EAE) T3-T4: 63 d (starting 5 wks pre-EAE)	*CD*: ↓ mortality in T1-T4 except T4 males (↑); – EAE onset, peak, mean CDS, infiltration of MNCs *IM: –* MBP IgG *MM*: NA *MC*: NA
Kobayashi et al.^74^	UN	F SJL & C57BL6 mice 7 wko *n* = 15/g	PLP-induced EAE (SJL) & MOG-induced EAE (C57BL6)	**T**: *L. casei* YIT 9029, 0.6–1.2 × 10^9^ CFU o.g. **C**: 0.2 mL saline/peptone	Proph & Thera	Daily for 50 (SJL) or 29 (C57BL6) d (both starting 1 wk pre-EAE)	*CD*: ↓ EAE CS on ds 12, 29, & 30 (SJL); – EAE onset, peak score, MNC infiltration, white matter demyelination, or neutrophil infiltration (SJL & C57BL6) *IM*: ↓ % CD8 + T cells in spleen (SJL), ↑ IL-10, % Tregs in spleen, IL-17, IFN-γ (SJL) *MM*: NA *MC*: NA
Lavasani et al.^48^	UN	F C57BL6 (WT & IL-10-/-) mice 8–10 wko *n* = 3–18/g	MOG-induced EAE	**T1**: Proph *L. paracasei* DSM 13434 **T2**: Proph *L. plantarum* DSM 15312 **T3**: Proph *L. plantarum* DSM 15313 **T4**: Proph *L. paracasei* PCC 101 **T5**: Proph *L. delbrueckii* subsp. bulgaricus DSM 20081 Each 5 mL of 10^9^ CFU orally until EAE, then 200 µL of 10^9^ CFU o.g. **T6**: Thera T1 **T7**: Thera Lacto-mix (T1-T3) **T8**: HK T7 **T9**: T7 in IL-10-/- mice Each 200 µL of 10^9^ CFU o.g. **C**: saline	Proph & Thera	Daily for 37 d (starting 12 d pre-EAE) or every other day for 20 d (starting 2 wks post-EAE onset)	*CD*: – EAE progression (T1-T5,T8); delayed EAE onset and ↓ CS (T1-T3, T7); therapeutic effects of Lacto-mix absent in T9 *IM*: ↓ T cell proliferation (T1-T3) ↓ CD4 + T cells (T1,T3),, ↓ IFN-γ, TNF-α (T1); ↑ IL-4, IL-10, TGF-β (T1); ↓ CNS inflammation, CD4 + T cell infiltration, IFN-γ, TNF-α, IL-17, and IL-17-producing CD4 + T cells (T7); ↑ IL-10, IL-10-producing CD4 + T cells, Tregs, Foxp3+ cells in brain, mLN, spleen (T7) *MM*: NA *MC*: EAE suppressed in recipient mice (mLN cells from T7) & depletion of CD4+ CD25 + T cells from mLN cells reverses suppression
Sanchez et al.^49^	UN	M & F C57BL6 (WT, CD45.1, CD45.2, GF, TLR-2-/-, & TLR-9-/-) mice & SJL mice 9–10 wko *n* ≥ 10/g	MOG-induced EAE (C57BL6) & PLP-induced EAE (SJL)	**T1**: Proph *L. paracasei* ATCC 27092 in C57BL6 mice **T2**: Proph HK T1 in C57BL6 mice **T3**: Proph *L. paracasei* DSM 2649 in C57BL6 mice **T4**: Proph *L. paracasei* ATCC 11582 in C57BL6 mice **T5**: Proph *L. paracasei* ATCC 334 in C57BL6 mice **T6**: Proph *L. paracasei* DSM 5622 in C57BL6 mice **T7**: T2 in CD45.1 C57BL6 donor mice AT into CD45.2 C57BL6 mice **T8**: T2 in TLR-2-/- C57BL6 mice **T9**: T2 in TLR-9-/- C57BL6 mice **T10**: T2 in WT C57BL6 mice GMT into GF C57BL6 mice **T11**: Thera T2 in SJL mice Each 10^9^ CFU o.g. **C**: PBS, MRS medium, or T1-conditioned medium	Proph & Thera	Daily for 34–39 d (starting 2 wks pre-EAE) or daily for 39 d (starting 21 dpi) **T10**: daily for 28 d (starting 70 d pre-EAE) and GMT at 42 d pre-EAE	*CD*: ↓ EAE CS (T1-T6); ↓ EAE incidence (T1-T6); ↓ demyelination, infiltrating macrophages & lymphocytes in brain & spinal cord, and severity of subsequent relapses (T1-T2); ↑ EAE CS w/ T1-conditioned media; – BBB & BSCB permeability (T2) *IM: –* prop. CD4+ IFN-γ + T cells, CD4+ IL-17A+ T cells, or Tregs in CNS (T1-T2); ↓ CCL3, CCL4, CXCL5, CXCL13 (T2) *MM*: ↓ EAE incidence (T10); – EAE CS (T10) *MC*: EAE suppression and ↓ demyelination eliminated in TLR2-/- mice; – EAE incidence or severity in AT recipient mice of T2
Yamashita et al.^67^	UN	F SJL mice 5 wko *n* = 10/g	PLP-induced EAE	**T**: HK *L. helveticus* SBT2171, 1 mg i.p. **C1**: 1 mg PBS i.p. **C2**: PBS, no EAE (n = 3)	Proph & Thera	3×/wk for 3 wks (pre-EAE), daily for 42 dpi	*CD*: ↓ EAE incidence, CS, enlargement of inguinal LNs, infiltrating MNCs *IM*: ↓ # T_H_17, T_H_1, & CD4 + T cells in spinal cord, IL-17; ↓ IL-6, TGF-β, Foxp3+, IFN-γ in inguinal LNs; – IL-10 in inguinal LNs, – Ccl20 in spinal cord, CCR2, CCR4, & CCR6 on T_H_17 cells in dLNs *MM*: NA *MC*: NA
Libbey et al.^54^	UN	M C57BL6 mice 4 wko *n* = 15/g	MOG-induced EAE	**T1**: r*E. coli* Nissle 1917, 1.31 × 10^9^ CFU o.g. single gavage day **T2**: r*E. coli* Nissle 1917, 1.19 × 10^9^ CFU + 1.25 × 10^9^ CFU o.g. double gavage **C1**: 70 µL PBS **C2**: r*E. coli* Nissle 1917, no EAE	Proph	1–2 admins 3 or 7 d pre-EAE Follow for 35–36 dpi	*CD*: ↓ survival & – CS, onset, incidence, CDS, maximal score, meningitis, demyelination, PVC (T1); ↓ CS, weight loss, PVC in T2; ↑ survival (T2); – EAE onset, incidence, CDS, maximal score, meningitis, demyelination (T2) *IM*: ↑ microglia, Tregs, IFN-γ, IL-27 (T1); ↓ CNS-derived cells, microglia, CD8 + T cells, CD4 + T cells, T_H_1 cells, Tregs in brain (T2); ↑ T_H_17 cells (T2) *MM*: NA *MC*: NA
Secher et al.^55^	UN	M C57BL6 mice 8–12 wko *n* = 30–40/g	MOG-induced EAE	**T1**: *E. coli* Nissle 1917 **T2**: archetypal K12 *E. coli* MG1655 Each 10^8^ CFU o.g. **C**: PBS	Proph & Thera	Daily for 37 d (starting 7 d pre-EAE)	*CD*: ↓ EAE CS, mortality, incidence, CDS, maximal score; – onset; ↓ colon & ileum permeability *IM*: ↓ total CD4+ and MOG-specific CD4 + T cells in spinal cord, IFN-γ, GM-CSF, IL-17, TNF-α, IL-6; ↑ total CD4+ and MOG-specific CD4 + T cells in LNs, IL-10, CD4+ Foxp3+ cells from draining LNs *MM*: NA *MC*: ↑ expression of Reg3g, Reg3b, Claudin-8, ZO-1
Mestre et al.^43^	UN	F SJL mice 5–8 wko *n* = 5–10/g	TMEV-IDD	**T**: TMEV-IDD w/ Vivomixx^b^ 100 µL of 3 × 10^8^ CFU o.g. **C1**: Sham w/ Vivomixx^b^ **C2**: TMEV-IDD w/ vehicle **C3**: Sham w/ vehicle	Thera	3×/wk for 15 d (70–85 dpi)	*CD*: ↑ horizontal & vertical activity, latency to fall *IM*: ↓ CD4 + T cells, B cells, % Foxp3+ CD39+ and Foxp3-CD39 + T cells in spleen, IL-1B, IL-6; ↑ Bregs, IL-10; – TNF-α; microglia exhibited anti-inflammatory activation or ↓ proinflammatory activity *MM*: ↓ rel. abund. of *Anaerostipes, Dorea, Oscillospira, Enterobacteraceae, Ruminococcus, Bilophila*, ↑ rel. abund. of *Bacteroides, Odoribacter, Lactobacillus, Sutterella*; ↑ acetate & butyrate in plasma *MC*: NA
Calvo-Barreiro et al.^44^	Low	F C57BL6 OlaHsd mice 8 wko *n* = 17–20/g	MOG-induced EAE	**T1**: Lactibiane iki^c^, 1.6 × 10^9^ CFU o.g. once daily **T2**: T1 twice daily **T3**: Vivomixx^b,^ 9 × 10^9^ CFU o.g. once daily **T4**: T3 twice daily **C1**/**C2**: water o.g. once or twice daily **C3**: untreated EAE **C4**: normal control	Thera	1–2× daily for 18–22 d (13–16 or 12–15 dpi)	*CD*: ↓ CS w/ T1/T2 but not T3/T4 (dose-response observed); ↓ % demyelination, T cell inflammatory infiltrate density, & axonal damage (T1-T4); – intestinal permeability or microglia or astrocyte reactivity; improved motor coordination *IM*: ↓ ASP w/ T1/T2 but not T3/T4, % peripheral plasma cells (T1/T2); ↑ Tregs (T1/T2) *MM*: – alpha or beta diversity; ↑ rel. abund. of *Lachnoclostridium* and *Bifidobacterium* (T1/T2), *Streptococcus (*T3/T4); *Atopobiacaeae* & *Bifidobacterium* assoc. with ↓ accumulated EAE scores *MC*: 4x↓ expression of Th17 txn factor RORγt in spinal cord T1/T2
Kwon et al.^69^	UN	C57BL6 mice 6–8 wko *n* = 10/g	MOG-induced EAE	**T1**: Proph IRT5^d^ **T2**: Thera IRT5^d^ Each 5 × 10^8^ CFU o.g. **C**: PBS	Proph & Thera	Daily for 3 wks (pre-EAE) or 16 d (starting 12 dpi)	*CD*: ↓ EAE incidence, CS, lymphocytes, Gr1+ and CD11b+ monocytes, and CD4 + T cells in spinal cord (T1); delayed EAE onset & ↓ EAE CS (T2) *IM*: ↓ ASP, IFN-γ, TNF-α, IL-17; ↑ IL-4, IL-10, CD4+ Foxp3+ Tregs from spinal cord *MM*: NA *MC*: NA
McMurran et al.^45^	Low	F C57BL6 mice 13 mo *n* = 3–5/g	Cuprizone (5 wks)	**T**: VSL#3^e^, 1.35 × 10^9^ CFU o.g. **C**: autoclaved water	Proph & Thera	Daily for 7 wks (starting 4 wks pre-lysolecithin injection)	*CD*: – remyelination *IM*: ↑ SCFAs in feces & serum, inflammatory response at 5 dpi, density of CD68+ activated microglia & infiltrating macrophages, # of ODCs at 5 dpi; – ODC response at 14 dpi, inflammatory response at 14 dpi *MM*: NA *MC*: NA
Ezendam et al.^51^	UN	M & F Lewis rats 2 wko n = 4–8/g	gpMBP-induced EAE	**T**: *B. animalis* w/ EAE, 200 µL of 1 × 10^9^ CFU o.g. **C1**: *B. animalis* w/ no EAE **C2**: 200 µL saline w/ EAE **C3**: 200 µL saline w/ no EAE	Proph & Thera	Daily for ~60 d (starting 5 wks pre-EAE)	*CD*: ↑ weight gain for M; shorter EAE duration for M; – EAE onset, duration, or CDI for F; – EAE onset or CDI for M *IM*: NA *MM*: NA *MC*: NA
Ezendam & van Loveren^72^	High	M & F Lewis rats 2 wko *n* = 4–8/g	gpMBP-induced EAE	**T;** *L. casei* strain Shirota, 500 µL of 1–2 × 10^9^ CFU o.g. **C**: 500 µL saline/peptone	Proph & Thera	Daily for ~9 wks (starting 5 wks pre-EAE)	*CD*: – EAE onset, CS, CDI for M & F; slightly longer duration for F; ↑ EAE incidence *IM*: NA *MM*: NA *MC*: NA
Johanson et al.^76^	UN	F C57BL6 mice 8 wko n = 10/g	MOG-induced EAE	**T**: *L. reuteri* ATCC 2327 ad libitum **C**: MRS broth	Thera	Daily for 20 d	*CD*: ↓ average CS, – weight loss *IM*: NA *MM*: ↑ Lactobacillus 16S V3-V4 amplicon abundance *MC*: NA
Abdurasulova et al.^66^	UN	F Wistar rats 3 mo *n* = 26–35/g	homologous SCH-induced EAE	**T1**: *E. faecium* LMG *P*-27496 L3 probe, 0.5 mL o.g. **T2**: Glatiramer acetate, 0.2 mL s.c. **C1**: saline, 0.5 mL o.g. **C2**: saline, 0.2 mL s.c.	Thera	Daily for 15 d (starting 2 dpi)	*CD*: ↓ prevalence, CDS, CS, mortality (T1/T2); delayed onset and shorter duration (T1/T2); T1 outperformed T2 in prevalence, duration, and CDS *IM*: ↓ CD4+ CD25+ Foxp3+ Tregs during peak and recovery phases, CD4 + T cells during peak; ↑ CD4+ Tcells during inductive, CD4+ CD25+ Foxp3- Tregs during peak, CD8 + T cells during peak & recovery *MM*: NA *MC*: NA

All findings are reported with respect to control group(s) unless otherwise indicated.

**^b^Vivomixx** = *L. paracasei* DSM 24734, *L. plantarum* DSM 24730, *L. acidophilus* DSM 24735, *L. delbrueckii* subsp. *bulgaricus* DSM 24734, *B. longum* DSM 24736, *B. infantis* DSM 24737, *B. breve* DSM 24732, *S. thermophilus* DSM 24731.

**^c^Lactibiane iki** = *B. lactis* LA 304, *L. acidophilus* LA 201, *L. salivarius* LA 302.

**^d^IRT5 **= *L. casei, L. acidophilus, L. reuteri, B. bifidum, S. thermophilus.*

**^e^VSL#3** = see ^b^Vivomixx.

**Key**: ↓ decreased; ↑ increased; – no change or no difference compared to control; NA, not applicable to this study.

**Abbreviations**: ROB, risk of bias; MS, multiple sclerosis; UN, uncertain; M, male; *F*, female; *wk/wks/wko*, week/weeks/weeks old; mo/mos, month/months; g, group; WT, wild-type; IL, interleukin; GF, germ-free; TLR, toll-like receptor; EAE, experimental autoimmune encephalomyelitis; MOG, myelin oligodendrocyte glycoprotein; PLP, proteolipid protein; MBP, myelin basic protein; gp, guinea pig; SCH, spinal cord homogenate; GID, gliotoxin-induced demyelination; TMEV-IDD, Theiler’s murine encephalomyelitis virus-induced demyelinating disease; CFU, colony-forming units; T, treatment group; C, control; o.g., oral gavage; IU, international units; w/w, weight/weight; HK, heat-killed; -/-, deficient; AT, adoptive transfer; GMT, gut microbiome transfer; i.p., intraperitoneally; PBS, phosphate-buffered saline; Proph, prophylactic; Thera, therapeutic; admins, administrations; dpi, days post immunization; CD, clinical disease; IM, immune/metabolic; MM, microbiome/metabolome; MC, mechanistic/correlative; CDB, clinical disease burden; CS, clinical score; CDI, clinical disease index; MNC, mononuclear cell; IgG, immunoglobulin G; ASP, antigen-specific proliferation; IFN-γ, interferon gamma; TGF-β, transforming growth factor beta; CDS, cumulative disease score; rel. abund., relative abundance; TNF-α, tumor necrosis factor alpha; CNS, central nervous system; LN, lymph node; BBB, blood–brain barrier; BSCB, blood spinal cord barrier; PVC, perivascular cuffing; GM-CSF, granulocyte monocyte colony-stimulating factor; txn, transcription; *SCFA*, short-chain fatty acid; ODC, oligodendrocyte; *abs*., absolute

**Table 4.** Animal studies of commensal therapies: summary of study characteristics and major findings

Study	ROB	Sample	MS Model	Intervention	Timeline	Duration	Major Findings
Montgomery et al.^37^	Low	M & F C57BL6 & PWD mice 4 wko	2× MOG-induced EAE	**T**: *L. reuteri* isolated from PWD cecal contents (100 µL of 10^9^ CFU o.g.) w/ 100 µL cryopreserved C57BL6 cecal microbiota **C**: 200 µL cryopreserved C57BL6 cecal microbiota	Proph	4 wks (one initial admin)	*CD*: ↑ CDS & freq. of infiltrating CD4 + T cells in spinal cord; – freq. of infiltrating CD8 + T cells in spinal cord *IM*: ↑ freq. of GM-CSF- and IFN-γ- producing CD4+ & CD8 + T cells *MM*: NA *MC*: NA
Chen et al.^65^	UN	F C57BL6 mice 3–4 wko *n* = 8/g	2× MOG-induced EAE	**T1**: *C. butyricum* GDBIO1501, 5 × 10^6^ CFU/mL o.g. **T2**: norfloxacin, 5 mg/kg o.g. **C1**: PBS w/ EAE **C2**: normal control	Proph	Daily for 3 wks pre-EAE	*CD*: ↓ daily CS, lymphocyte infiltration, demyelinating plaques in lumbar spinal cord *IM*: ↓ T_H_17 cells in CNS, LN, colon, spleen, & small intestine, IFN-γ-producing CD4 + T cells in spleen; – IL-17A+IFN-γ+ CD4 + T cells; ↑ differentiation of Tregs *MM*: ↑ # of OTUs, abundance, diversity, and rel. abund. of *Prevotella*, Bacteriodetes; ↓ rel. abund. of Firmicutes, Desulfovibroneceae, *Ruminococcus* *MC*: ↓ phosphorylation of p38 MAPK, ERK1/2, and JNK in lumbar spinal cord
Mangalam et al.^56^	UN	M & F HLA-DR3.DQ8 transgenic mice 8–12 wko *n* = 5–11/g	PLP-induced EAE	**T1**: Proph *P. histicola*, 10^8^ CFU/mL o.g. **T2**: Proph *P. melaninogenica*, 10^8^ CFU/mL o.g. **T3**: Proph *C. sputigena*, 10^8^ CFU/mL o.g. All isolated from duodenum of celiac disease patients **T4**: Proph mouse-specific *E. coli*, 10^8^ CFU/mL o.g. **T5**: T1 w/ Abx-depleted flora **T6**: Thera T1 **T7**: HK T6 **T8**: T6 cell-free supernatant **T9**: T6 10^7^ CFU **T10**: T6 10^9^ CFU **C**: medium	Proph & Thera	Every other day for 2 wks (starting 7 d pre-EAE or 7 dpi) Abx depletion for 3 wks AT at 5 dpi	*CD*: ↓ EAE incidence, CDS, regions of brain & spinal cord inflammation and demyelination, BBB permeability, CNS cellular infiltration (T1); earlier onset (T1); T1 restored gut permeability *IM*: ↓ IL-23, IL-12, IFN-γ, IL-17, CD4 + T cells, IFN-γ- and IL-17-expressing CD4 + T cells (T1); ↑ IL-10, TGF-β, CD4+ CD25+ Foxp3+ Tregs (T1); ↑ IL-10 (T2) *MM*: ↑ rel. abund. of *Prevotella, Lactobacillus, Sutterella* (T1), resembling pre-EAE states *MC*: Milder EAE (T5); ↑ EAE incidence (T7/T8); ↑ EAE suppression (T6 vs T9/T10); ↓ EAE incidence in AT recipient mice of T1
Shahi et al.^57^	UN	M & F HLA-DR3.DQ8 double transgenic mice & C57BL6 mice 8–12 wko *n* ≥ 7/g	PLP-induced EAE (HLA) and MOG-induced EAE (C57BL6)	**T1**: Proph *P. histicola*, 10^8^ CFU o.g. in HLA mice **T2**: Proph Copaxone, 2 mg s.c. in HLA mice **T3**: T1+ T2 **T4**: T1 in C57BL6 mice **T5**: T2 in C57BL6 mice **T6**: T3 in C57BL6 mice **T7**: Thera T1 **T8**: Thera T2 **T9**: Thera T3 **C**: PBS or TSB media	Proph & Thera	Every other day for 2 wks (starting 7 d pre-EAE or 7 dpi) T3, T6, T9 = T1 & T2 on alternating days	*CD*: ↓ average daily scores & CDS (T2,T3,T4-T6,T7-T9); delayed EAE onset (T9); ↓ inflammation, demyelination (T1-T3); effects of T3/T6/T9 not more pronounced *IM*: ↓ IL-17+ CD4+ & IFN-γ+ CD4 + T cells in brain & spinal cord (T1,T3); ↑ CD4+ Foxp3+ Tregs in splenocytes and GALT (T1,T3); – IL-10-producing CD4 + T cells (T1-T3) *MM*: ↑ rel. abund. *Lactobacillus* (T1,C), ↓ (T2,T3) *MC*: NA
Shahi et al.^58^	UN	M & F HLA-DR3.DQ8 double transgenic mice 8–12 wko *n* ≥ 12/g	PLP-induced EAE	**T1**: Proph *P. histicola*, 10^8^ CFU o.g **T2**: Proph IFNβ, 10,000 IU **T3**: Proph T1+ T2 **T4**: Thera T1 **T5**: Thera T2 **T6**: Thera T3 **C**: TSB media	Proph & Thera	Every other day for 2 wks pre-EAE (7 doses) or starting 7 dpi (7 doses)	*CD*: ↓ average daily score & CDS (T4-T6), inflammatory cellular infiltration into brain & spinal cord (T4,T6), spinal cord tissue and meningeal/stratum regions of brain (T4-T6); no additive effects for T6 vs T4/T5 *IM*: ↓ Iba-1+ microglia & GFAP+ astrocytes in brain & spinal cord white matter, CD4+ IL-17+ & CD4+ IFN-γ + T cells (T1-T3); ↑ CD4+ Foxp3+ Tregs, IL-10-producing CD4 + T cells (T1,T3) *MM*: NA *MC*: NA
Takata et al.^70^	UN	F C57BL6 mice & SJL mice 6 wko *n* = 17/g	MOG-induced EAE (C57BL6) and PLP-induced EAE (SJL)	**T1**: HK *P. acidilactici* R037 in C57BL6 mice, 20 mg/mL o.g. **T2**: HK *P. acidilactici* R037 in water in SJL mice, 0.8 mg/mL orally **C1**: PBS, o.g. **C2**: PBS in water, orally	Proph & Thera	Daily for 36 d (starting 2 wks pre-EAE)	*CD*: ↓ CS (T1,T2); ↓ infiltrating MNCs (T1); delayed EAE onset (T2) *IM*: ↓ IL-17 & IFN-γ in splenocytes & draining LNs; ↑ IL-10 & CD4+ IL-10 + T cells in mesenteric LNs and splenocytes *MM*: NA *MC*: NA
Miyauchi et al.^38^	Low	F GF C57BL6 mice 5–7 wko *n* = 5–10/g	MOG-induced EAE	**T1**: OTU0001 (*L. reuteri*, 100% 16S rRNA match to strains H4 & LMG 18238) **T2**: OTU0002 (*Allobaculum*) Both isolated from small intestine of specific PF mice and o.g. at 5–7 wko for stable colonization. **T3**: T1 + T2 co-colonized **T4**: T3 with *urvA*-deficient *L. reuteri* **C**: naïve GF	Proph	One admin at 5–7 wko	*CD*: ↑ CS (T2,T3); – CS (T1); ↓ CS, EAE incidence (T4); ↑ demyelination, spinal cord infiltration, EAE incidence (T3) *IM*: ↑ IL-17A, T_H_17 cells in lamina propria of small intestine & splenocytes (T2); ↑ Tregs in small intestine (T2); ↑ T_H_17 cells (T3); – IL-17A, T_H_17 cells, Tregs in small intestine (T2/T3); – T_H_17 cells in small intestine (T1/T4) *MM*: – rel. abund. in small intestine (T2/T3); – rel. abund. in small intestine (T1/T4) *MC*: ↑ expression of *Saa1, Saa2, Il23a, Il12b, Csf2, Il23r* in small intestine (T2); – expression of *Saa1, Saa2, Il23a* in small intestine (T2/T3)
Ochoa-Reparaz et al.^62^	UN	F SJL mice 6 wko *n* = 6–8/g	PLP-induced EAE	**T1**: 1 wk Abx, recolonize w/ WT *B. fragilis* NCTC 9343 **T2**: 1 wk Abx, recolonize w/ PSA-deficient *B. fragilis* Both at 10^10^ CFU in 200 µL sterile PBS o.g. **C1**: 1 wk Abx only **C2**: sham (no Abx, receive PBS)	Proph	30 d (one initial admin)	*CD*: ↓ CS, CDS (T1,C1); delayed EAE onset (T1,C1); – EAE incidence *IM*: ↓ Tbet (C1), IFN-γ (C1), RORγt (T1,C1), IL-17 (T1,C1); ↑ GATA-3 (T1,C1), IL-10 (T1,C1), SMAD-3 (T1), IL-13 (C1), freq. Foxp3+ CD25+ CD4 + T cells in cervical LNs (C1); ↑ IL-17, RORγt, Tbet and ↓ GATA-3, IL-10, IL-13 (T2); ↓ conversion of CD103+ DCs to Foxp3+ Tregs (T2) *MM*: # of detectable bacteria after Abx restored (T1,T2) (colonization confirmed) *MC*: Depletion of CD25 + T cells eliminated protective effects (C1,T1); ↓ CS & ↑ IL-10 in AT recipient mice (T1 only)
Liu et al.^35^	UN	F C57BL6 mice 6–8 wko *n* = 23–28/g	MOG-induced EAE	**T1**: *Akkermansia muciniphila* ATCC BAA-835 **T2**: *E. coli* K-12 Strain #7296 **C**: medium	Thera	Daily for 7 d (starting 11 dpi)	*CD*: ↓ CS, demyelination, axonal loss (T1) *IM: ↑* MOG-specific Foxp3+ Tregs and total Tregs in spleen (T1); ↑ Foxp3+ Tregs from DCs (T1); – direct Foxp3+ Treg induction (T1 vs T2); ↓ IL-6, IL-1b expression in DCs (T1), – TGF-β expression (T1/T2) *MM*: NA *MC*: NA

All findings are reported with respect to control group(s) unless otherwise indicated.

**Key**: ↓ decreased; ↑ increased; – no change or no difference compared to control; NA, not applicable to this study.

**Abbreviations**: ROB, risk of bias; MS, multiple sclerosis; UN, uncertain; M, male; F, female; wk/wks/wko, week/weeks/weeks old; g, group; EAE, experimental autoimmune encephalomyelitis; MOG, myelin oligodendrocyte glycoprotein; PLP, proteolipid protein; T, treatment group; C, control; CFU, colony-forming units; o.g., oral gavage; PBS, phosphate-buffered saline; s.c., subcutaneous; Abx, antibiotics; HK, heat-killed; IFNβ, interferon beta; IU, international unit; OTU, operational taxonomic unit; PF, pathogen-free; GF, germ-free; WT, wild-type; PSA, polysaccharide A; Thera, therapeutic; Proph, prophylactic; admin; administration; dpi, days post immunization; CD, clinical disease; IM, immune/metabolic; MM, microbiome/metabolome; MC, mechanistic/correlative; CDS, cumulative disease score; freq., frequency; GM-CSF, granulocyte monocyte colony-stimulating factor; IFN-γ, interferon gamma; CS, clinical score; CNS, central nervous system; LN, lymph node; IL, interleukin; rel. abund., relative abundance; BBB, blood–brain barrier; AT, adoptive transfer; GALT, gut-associated lymphoid tissue; DC, dendritic cell; TGF-β, transforming growth factor beta; MNC, mononuclear cell.

**Table 5.** Bradford Hill criteria evaluation of probiotic and commensal therapies

Therapy	Study	Study Type	ROB	Strain Used	Treatment Effect	BH Score
VSL#3/ Vivomixx/ LBS	Mestre et al.^43^	Animal TMEV-IDD Thera	UN	*L. paracasei* DSM 24734, *L. plantarum* DSM 24730, *L. acidophilus* DSM 24735, *L. delbrueckii* ssp. bulgaricus DSM 24734, *B. longum* DSM 24736, *B. infantis* DSM 24737, *B. breve* DSM 24732, *S. thermophilus* DSM 24731	Improved	**9**
	Calvo-Barreiro et al.^44^	Animal EAE Thera	Low		Improved	
	McMurran et al.^45^	Animal Cuprizone Proph & Thera	Low		No change	
	Tankou et al.^46^	Human Prospective Cohort	High		Improved (immunologically)	
	Tankou et al.^47^	Human Prospective Cohort	High		Improved (immunologically & microbiologically)	
*L. paracasei*	Lavasani et al.^48^	Animal EAE Proph & Thera	UN	DSM 13434, PCC 101	Improved	**7**
	Sanchez et al.^49^	Animal EAE Proph & Thera	UN	ATCC 27092 (live & heat-killed), ATCC 11582, DSM 2649, DSM 5622, ATCC 334	Improved	
*B. animalis*	Salehipour et al.^50^	Animal EAE Thera	Low	PTCC 1631	Improved	**7**
	Ezendam et al.^51^	Animal EAE Proph & Thera	UN	Not specified	No change	
	Goudarzvand et al.^52^	Animal GID Thera	UN	Susbp. lactis B94	No change	
	Consonni et al.^53^	Animal EAE Proph & Thera	Low	Subsp. lactis BB12	Slightly improved	
	Consonni et al.^53^	Animal EAE Proph & Thera	Low	Subsp. lactis LMG S-28195	Slightly improved	
*E. coli*	Libbey et al.^54^	Animal EAE Proph	UN	Nissle 1917 recombinant strain for ampicillin resistance (pGEN-MCS)	Improved	**7**
	Secher et al.^55^	Animal EAE Proph & Thera	UN	Nissle 1917	Improved	
*P. histicola*	Mangalam et al.^56^	Animal EAE Proph & Thera	UN	Isolated from duodenum of celiac disease patients	Improved	**7**
	Shahi et al.^57^	Animal EAE Proph & Thera	UN	Isolated from duodenum of celiac disease patients	Improved	
	Shahi et al.^58^	Animal EAE Proph & Thera	UN	Isolated from duodenum of celiac disease patients	Improved	
*L. plantarum*	Lavasani et al.^48^	Animal EAE Proph & Thera	UN	DSM 15312, DSM 15313	Improved	**6**
	Salehipour et al.^50^	Animal EAE Thera	Low	A7	Improved	
	Goudarzvand et al.^52^	Animal GID Thera	UN	Not specified	No change	
	Maassen et al.^59^	Animal EAE Proph	UN	NCIB 8826, 14917	No change	
Lacto-mix	Lavasani et al.^48^	Animal EAE Proph & Thera	UN	*L. plantarum* DSM 15312, *L. plantarum* DSM 15313, *L. paracasei* DSM 13434	Improved	**6**
*L. crispatus & L. rhamnosus*	Consonni et al.^53^	Animal EAE Proph & Thera	Low	*L. crispatus* LMG *P*-23257 & *L. rhamnosus* ATCC 53103	Improved	**6**
*B. animalis & B. animalis*	Consonni et al.^53^	Animal EAE Proph & Thera	Low	*B. animalis* subsp. lactis BB12 & *B. animalis* subsp. lactis LMG S-28195	Improved	**6**
*L. acidophilus, L. casei, L. fermentum, & B. bifidum*	Kouchaki et al.^60^	Human RCT	Low	Not specified	Improved	**5**
	Tamtaji et al.^61^	Human RCT	Low	Not specified	Slightly improved (immunologically)	
*B. fragilis*	Ochoa-Reparaz et al.^62^	Animal EAE Proph	UN	NCTC 9343	Improved	**5**
*L. crispatus*	Consonni et al.^53^	Animal EAE Proph & Thera	Low	LMG *P*-23257	Improved	**4**
*L. rhamnosus*	Consonni et al.^53^	Animal EAE Proph & Thera	Low	ATCC 53103	Improved	**4**
Lactibiane iki	Calvo-Barreiro et al.^44^	Animal EAE Thera	Low	*B. lactis* LA 304, *L. acidophilus* LA 201, *L. salivarius* LA 302	Improved	**4**
Protexin	Rahimlou et al.^63^	Human RCT	Low	*B. subtilis* PXN 21, B. bifidum PXN 23, B. breve PXN 25, *B. infantis* PXN 27, *B. longum* PXN 30, *L. acidophilus* PXN 35, *L. rhamnosus* PXN 54, *L. helveticus* PXN 45, *L. salivarius* PXN 57, *L. lactis* ssp. lactis PXN 63, *S. thermophilus* PXN 66, *L. casei* PXN 37, *L. delbrueckii* ssp. bulgaricus PXN 39, *L. plantarum* PXN 47	Slightly improved	**4**
*L. plantarum, L. casei, L. reuteri, L. fermentum, B. infantis, & B. lactis*	Salami et al.^64^	Human RCT	Low	Not specified	Improved	**4**
*L. plantarum & B. animalis*	Salehipour et al.^50^	Animal EAE Thera	Low	*L. plantarum* A7, *B. animalis* PTCC 1631	Improved	**4**
*C. butyricum*	Chen et al.^65^	Animal EAE Proph	UN	GDBIO1501	Improved	**4**
*Allobaculum*	Miyauchi et al.^38^	Animal EAE Proph	Low	Isolated from content of small intestines of specific PF mice	Slightly worsened	**4**
*L. murinus*	Maassen et al.^59^	Animal EAE Proph	UN	CNRZ	Improved	**3**
*E. faecium*	Abdurasulova et al.^66^	Animal EAE Thera	UN	LMG *P*-27496	Improved	**3**
*L. helveticus*	Yamashita et al.^67^	Animal EAE Proph & Thera	UN	SBT2171	Improved	**2**
*B. breve*	Kobayashi et al.^68^	Animal EAE Proph & Thera	UN	Yakult	Slightly improved (F) Worsened (M)	**2**
IRT5	Kwon et al.^69^	Animal EAE Proph & Thera	UN	*L. casei*, *L. acidophilus*, *L. reuteri*, *B. bifidum*, *S. thermophilus*	Improved	**2**
*P. acidilactici*	Takata et al.^70^	Animal EAE Proph & Thera	UN	R037	Improved	**2**
*A. muciniphila*	Liu et al.^35^	Animal EAE Thera	UN	ATCC BAA-835	Improved	**2**
*L. casei*	Maassen et al.^59^	Animal EAE Proph	UN	393	Slightly improved	**1**
	Gharehkhani Digehsara et al.^71^	Animal Cuprizone Proph & Thera	UN	Not specified	Improved	
	Ezendam & van Loveren^72^	Animal EAE Proph & Thera	High	Shirota	Slightly worsened	
	Baken et al.^73^	Animal EAE Proph & Thera	UN	Shirota	Worsened	
	Kobayashi et al.^68^	Animal EAE Proph & Thera	UN	Shirota	No change	
	Kobayashi et al.^74^	Animal EAE Proph & Thera	UN	YIT 9029	No change	
*L. reuteri*	He et al.^75^	Animal EAE Thera	UN	DSM 17938	Improved	**1**
	Johanson et al.^76^	Animal EAE Thera	UN	ATCC 2327	Improved	
	Maassen et al.^59^	Animal EAE Proph	UN	ML1	Worsened	
	Miyauchi et al.^38^	Animal EAE Proph	Low	Isolated from content of small intestines of specific PF mice 100% 16S rRNA match to strains H4 & LMG 18238 Also *uvrA*-deficient *L. reuteri*	Slightly worsened	
	Montgomery et al.^37^	Animal EAE Proph	Low	Isolated from PWD cecal contents	Worsened	
*L. delbrueckii* subsp. *bulgaricus*	Lavasani et al.^48^	Animal EAE Proph & Thera	UN	DSM 20081	No change	**1**

**Abbreviations**: ROB, risk of bias; BH, Bradford Hill; EAE, experimental autoimmune encephalomyelitis; Proph, prophylactic; Thera, therapeutic; RCT, randomized controlled trial; GID, gliotoxin-induced demyelination; TMEV-IDD, Theiler’s murine encephalomyelitis virus-induced demyelinating disease; UN, uncertain.

**Table 6.** Bradford Hill criteria (BHC) warranting most attention for future probiotic and commensal therapy studies

	Probiotic Therapy	Commensal Therapy
**BHC #**	1. Temporal relationship	2. Strength of relationship
	4. Replication of findings	3. Dose–response relationship
	6. Cessation of exposure	4. Replication of findings
	7. Specificity of association	6. Cessation of exposure
		8. Coherence between multiple approaches

**Abbreviation**: BHC, Bradford Hill criteria.

## Results

### A. Study characteristics

**Figure 1.**
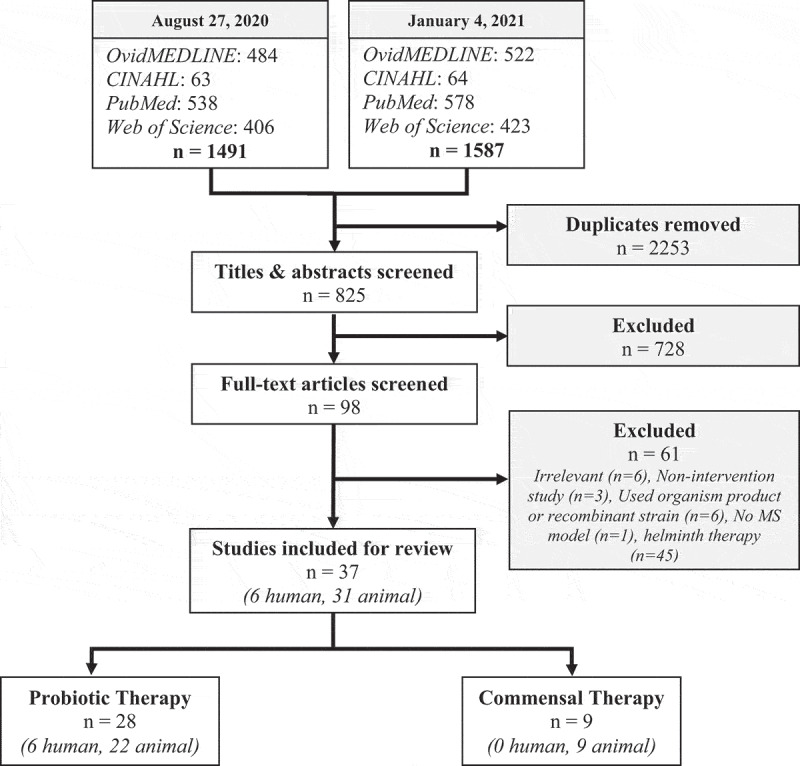
**Search strategies, selection criteria, and inclusion of articles**. Articles from four separate databases were identified and screened for inclusion. Note that helminth therapy studies were included in the search strategy but manually removed during the eligibility stage of the second search

The study characteristics and major findings of the included studies are summarized in Table 1**–**3. A total of 770 de-duplicated articles were found by the initial search, 55 additional articles were found by the second search, and one article^76^ was found by an author (DK) outside of the search strategy. A total of 37 studies^35,37,38^^43–76^ (6 human, 31 animal) were included for analysis in this review based on the stated selection criteria (see Methods) (Figure 1; Supp. File 1B). Of these 37 studies, 28 (6 human, 22 animal) investigated the effects of probiotic therapy and 9 (0 human, 9 animal) utilized commensal therapy. Studies were conducted between 1998 and 2020 in the following countries: USA, Iran, Japan, England, Netherlands, Spain, Russia, Italy, Sweden, France, China, and Republic of Korea.

#### Risk of bias

Overall, 10 studies (4 human, 6 animal) were deemed “high quality” based on the low risk of bias determined using the Cochrane ROB and SYRCLE tools (Supp. File 1D and 1 F). Most (*n* = 24) of the studies (0 human, 24 animal) were classified as “medium quality” due to study design limitations or failure to disclose randomization and/or blinding efforts. The remaining studies (*n* = 3) were classified as “low quality,” including two human studies that had important baseline characteristic differences between groups, substantial risk of confounding due to concurrent use of a DMT (glatiramer acetate), and compared the results to healthy controls rather than untreated MS patient controls^46,47^^,^; and one animal study that was not powered to perform statistical analysis lacked clarity regarding the control groups and did not explicitly state the use of randomization or blinding measures.^72^

#### Study design

Four human studies were structured as double-blind, placebo-controlled RCTs,^60,61,63,64^ and the remaining two were prospective cohort studies.^46,47^^,^ The animal models of MS included EAE (*n* = 27), cuprizone-^45,71^ or gliotoxin-induced demyelination,^52^ and Theiler’s murine encephalomyelitis virus-induced demyelinating disease (TMEV-IDD).^43^

#### Subjects

Human subjects were studied exclusively in the RRMS stage. Expanded disability status scores (EDSS) were all ≤4.5 as a maximum for inclusion. Male and female participants ranged from 18 to 60 y old. Animal subjects included both male and female rats (Lewis and Wistar) and mice (C57BL/6, SJL, and PWD/PhJ) ranging in age from 2 weeks to 13 months, with a mean of 7–8 weeks old. Various genetically modified C57BL/6 mice were used, including HLA-DR3.DQ8 transgenic^56,57,58^ and IL-10-,^48^ TLR-2-,^49^ and TLR-9-^49^ deficient strains.

#### Interventions

Probiotic therapy involved individual strains and various combinations (including Lacto-mix, Lactibiane iki, IRT5, Protexin, VSL3/VSL#3/Vivomixx/LBS) of *Lactobacillus spp*. including *L. casei, L. paracasei, L. plantarum, L. reuteri, L. delbrueckii* subsp. bulgaricus, *L. crispatus, L. rhamnosus, L. murinus, L. brevis, L. helveticus, L. lactis* subsp. lactis, *L. fermentum, L. acidophilus*, and *L. salivarius*, Bifidobactererium ssp. including *B. animalis, B*. B94, *B. breve, B. longum, B. bifidum, B. infantis, B. lactis, B. subtilis* as well as *E. coli* Nissle 1917, *E. faecium*, and *S. thermophilus*. All four human RCT studies utilized probiotic combinations at 2 × 10^9^ CFU/mL ranging from 12 weeks to 6 months duration, while the prospective cohorts used 3.6 × 10^12^ CFU/d for 2 months. The animal studies administered a mean of 10^9^ CFU/mL (range of 10^8^–10^10^ CFU/mL) for 2–7 weeks duration. Probiotics were administered prophylactically,^54,59^^,^ therapeutically,^43,44,46,47,50,52,60,61,63,64,66,75,76^ or both.^45,51,55,69,72^^,^

There were no human studies using commensal therapy identified. Animal studies used *Prevotella histicola* (*P. histicola*),^56,57,58^ *P. acidilactici*,^70^*C. butyricum,*^65^*Akkermansia muciniphila* (*A. muciniphila*),^35^ *L. reuteri*^37,38^^,^ (by stable colonization), *Allobaculum,*^38^ and *B. fragilis*^62^ at a dosage ranging 5 × 10^6^ to 1 × 10^10^ CFU/mL for one administration or 2–5 weeks. Commensals were administered prophylactically,^37,38,62,65^ therapeutically,^35^ or both.^56,57,58,70^

#### Measurements and outcomes

The most common measurements for clinical parameters for human studies included EDSS, mental health and quality of life assessments (Beck Depression Inventory (BDI), General Health Questionnaire-28 (GHQ-28), Depression Anxiety Stress Scale (DASS), Fatigue Severity Scale (FSS), McGill Pain Questionnaire (MPQ)). Notably, none of the human studies assessed MRI lesions. For animal studies, clinical signs of motor disability and associated quantitative variables (EAE incidence, onset, duration, and clinical scores; motor function, coordination, and activity for other MS models), histopathology (demyelination, CNS infiltration), BBB and intestinal permeability, and weight loss. Cytokine analysis, oxidative stress/antioxidant markers, and immunophenotyping were the most commonly measured immune/metabolic indices. Microbiome and metabolome assessments were primarily measured using fecal microbiome analysis, and fecal/serum short-chain fatty acid (SCFA) production. Gene expression and adoptive transfer experiments comprised additional mechanistic and correlative findings.

## B. Probiotic therapy

### Major trends

The major findings of each probiotic therapy study included can be found in Tables 2 and 3 for human and animal studies, respectively. A qualitative summary of these studies is provided below, followed by a semi-quantiative ranked evaluation using BH criteria.

#### Clinical studies

Four of the human probiotic therapy studies included were double-blind, placebo-controlled RCTs, and thus all were classified as “high quality” studies.^60,61,63,64^ Probiotic therapy produced modest decreases in EDSS that, while sometimes statistically significant, were not found to be clinically significant based on the authors’ designation of an EDSS change of ≥1.0 point for levels less than 5.5 or ≥0.5 point for levels greater than 5.5.^60,64^ The impact on EDSS seemed more pronounced in the shorter, 12 week study, suggesting that the observed benefits may only be transient.^60^ Probiotic therapy did, however, lead to marked improvements in quality of life as measured through the BDI, GHQ-28, DASS, FSS, and MPQ assessments.^60,63,64^ The proinflammatory cytokines that were measured (IL-6, IL-8, and TNFα) were consistently reduced in the probiotic treatment groups, as were several oxidative stress markers (hs-CRP, MDA).^60,61,63,64^ Anti-inflammatory cytokines and antioxidants were measured, showing elevated IL-10 and plasma nitric oxide.^60,64^

Two additional prospective cohort studies used therapy with the probiotic mixture VSL#3.^46,47^ While neither study focused on clinical outcome, both found that VSL#3 elicited changes in the peripheral immune response consistent with an immune regulatory state, including phenotypic changes in monocytes and dendritic cells and decreased expression of the MS risk allele HLA-DQA1. Additionally, these studies found an increased relative abundance of *Lactobacillus, Bifidobacterium*, Streptococcus spp. in stool, which is consistent with the species that were administered in probiotic form in the VSL#3 formulation. Further, one study found an increased relative abundance of *Collinsela* and *Veillonellaceae* family members that are typically depleted in MS gut microbiomes, as well as a decreased relative abundance of *Akkermansia, Blautia*, and *Dorea* genera, which are typically enriched.^46^ Notably, these differences display an inverse relationship in MS patient cohort gut microbiomes, suggesting that VSL#3 may restore the MS-dysbiotic state.

#### Preclinical studies

The majority (26 out of 30) of animal studies used the EAE model. This is an important consideration, since it is a model driven by an autoimmune response, thus immunomodulation is the most likely mode of action for any effects on clinical disease. More studies investigated the effects of *Lactobacillus* spp.^48–50,52,53,59,67,68,71–76^ and probiotic combinations^43–45,48,50,53,69^ rather than *Bifidobacterium* spp.^51–53,68^ and *E. coli* Nissle 1917,^54,55^ and just a single study utilized *E. faecium*.^66^

Proportionally, *Lactobacillus* spp. tended to outperform *Bifidobacterium* spp. in reducing EAE incidence, onset, clinical score, duration, demyelination, immune cell infiltration, and motor activity. Positive clinical outcomes were observed in about 64% of *Lactobacillus* spp. studies (*n* = 14), 25% of *Bifidobacterium* spp. studies (*n* = 4), 100% of *E. coli* Nissle 1917 studies (*n* = 2), and about 81% of probiotic combination studies (*n*= 11), while *E. faecium* was shown to be at least as effective as the standard MS DMT glatiramer acetate.^66^ *L. casei* Shirota,^68,72,73^ and *B. animalis*^50–53^ were the least clinically successful therapies among the studies investigated, while *L. paracasei*,^48,49^ *L. plantarum*,^48,50^ and *E. coli* Nissle 1917^54,55^ appeared the most successful. Notably, despite disparate outcomes, VSL#3^45^ and Vivomixx^43,44^ contain the same probiotic formulation, although importantly each study used a different model: cuprizone-induced demyelination/remyelination vs. TMEV-IDD vs. EAE, respectively, with the former (cuprizone) lacking a strong immune-mediated component. Furthermore, *L. paracasei*^49^ and the combinations of *L. crispatus* and *L. rhamnosus*^53^ and *B. animalis* subsp. lactis strains^53^ were able to elicit clinical benefits whether administered live or heat-killed^,^ while Lacto-mix^48^ was only effective when live probiotic organisms were used. Five studies demonstrated a dose–response relationship,^44,48,50,53,54^ and three studies provided evidence for the combinatorial effects of probiotics.^43,45,69^ Only ~32% of the animal studies (*n* = 22) reported primarily no effect or exacerbation of clinical disease.^51,59,68,72–75^ It should also be noted that only four of the studies were classified as high quality, all of which reported positive results with probiotics.^44,45,50,53^

The majority of studies reported favorable secondary immunological findings, with elevated levels of anti-inflammatory cytokines (IL-10, IL-4, TGF-β)^43,48,50,53,55,59,69,71,74^ and CD4+ CD25+ FoxP3+ Tregs^44,50,54,55,69,74^ and reduced levels of proinflammatory cytokines (IL-17, IL-1, IL-6, TNF-α, IFN-γ),^43,48,50,53,55,67,69,71,75^ chemokines (CCL3, CCL4, CXCL5, CXCL13),^49^ and T_H_1 and T_H_17 cells.^43,54,55,67,69,74,75^ Multiple *Lactobacillus* spp. and probiotic combinations also demonstrated decreased antigen-specific T cell proliferation.^44,48,50,53,69^ Putatively beneficial microbiome changes included an increased relative abundance of *Firmicutes, Bacteriodetes, Proteobacteria* phyla and *Sutterella, Bifidobacterium, Streptococcus, Lactobacillus*, and *Prevotella* spp.^43,44,75^ Two studies measured SCFA production and found increased levels in both serum and feces.^43,45^ Four studies reported increased expression of T_H_2 and Treg regulators (*GATA3, Foxp3*),^50^ miR-25,^71^ antimicrobial peptides (Reg3g, Reg3b),^55^ and tight junction proteins (Claudin-8, ZO-I);^55^ and decreased expression of T_H_1 and T_H_17 regulators (*Tbet, RORγt)*,^44,50^ miR-155,^71^ and the *IDO* gene,^71^ a potential marker of MS/EAE relapses. Furthermore, Lavasani et al. performed an adoptive transfer experiment of CD4+ CD25 + T cells from mesenteric lymph nodes of the probiotic Lacto-mix group and found that the recipient mice had suppressed EAE symptoms and elevated IL-10 levels.^48^ These effects, however, were eliminated when tested in IL-10-deficient mice.^48^ Notably, Sanchez et al. also included an adoptive transfer experiment of splenic and mesenteric lymph node leukocytes of heat-killed *L. paracasei*–treated donor mice into recipient mice and found no such effects.^49^ Additional mechanistic findings included decreased intestinal barrier permeability^55^ and oligodendrocyte differentiation enhancement.^45^

### BHC scores and rankings

The BH score calculations and findings for probiotic therapy are detailed in Supplemental File 2, and summarized in Table 5. Given the large number of studies and treatments, we do not discuss each individually, but instead highlight and contrast some of the key findings below.

One probiotic treatment approach emerged as the most strongly supported (BH score = 9), namely the VSL#3 multi-species formulation, which was assessed in two human and three animal studies. Two out of three animal studies reported significant clinical improvement in the EAE model. The third study used the cuprizone demyelination model and reported a lack of clinical improvement, but some favorable histologic changes. The human studies did not measure clinical parameters, but reported immunological and microbiological changes that would be consistent with favorable immune modulation. Hence, this particular approach satisfied seven out of eight BH criteria (BHC #1, 3–8), with high evidence for replication (Table 5). In contrast, another combination treatment (*L. acidophilus, L. casei, L. fermentum*, and *B. bifidum*) was used in two high-quality human RCTs, but lacked supporting mechanistic and/or animal model studies (BH score = 5; Table 5). Additional promising probiotic treatments with high BH scores included *B. animalis, L. paracasei*, and *E. coli* Nissle 1917, each receiving a BH score of 7; and *L. plantarum*, Lacto-mix, *L. crispatus* & *L. rhamnosus*, and the *B. animalis* combination therapy, all of which received a BH score of 6.

The majority of the remaining microbial treatments were characterized by low BH scores, resulting from a paucity of studies, lack of mechanistic evidence, and/or presence of conflicting evidence. The latter is exemplified by *L. casei* (BH score = 1), which was examined in six animal studies, but showed evidence of disease exacerbation or lack of effect in four of those studies, resulting in a deduction of 2 points for BHC #4. We note that this interpretation is confounded by the fact that different strains/isolates were used across these different studies, highlighting the need for careful standardization and interpretation.

### BHC deficiencies

Using the Bradford Hill criteria, several therapies had considerable evidence for strength of relationship, dose–response relationship, biological plausibility, and coherence. Future studies should focus on strengthening these areas further by investigating dosing effects, establishing more direct evidence of MGBA involvement with more probiotic organisms and combinations, addressing alternative explanations, and repeating interventions in various contexts. Additionally, more evidence is needed to fulfill the remaining Bradford Hill criteria categories (BHC #s 1, 4, 6, and 7), including more before-and-after analyses, using a standardized protocol to facilitate comparisons across studies and research groups, and investigating cessation effects (Table 6). Strengthening the specificity of association by comparing the effects of live versus heat-killed organisms and their soluble products, and the inclusion of more mechanistic experiments is also recommended. Future studies and reviews should also consider the taxonomic reclassification of *Lactobacillus* spp. when referring to those probiotic organisms.^77^

Lastly, to move toward translational application, wherein probiotics are stringently defined as conferring a known benefit to human health, disease-specific usage should be assessed in well-powered RCTs to provide clinically relevant guidance. Notably, a defined benefit to MS patient health should not be limited to clinical outcome, but also include secondary parameters such as quality of life, since it is plausible that probiotic therapy may improve the well-known GI-associated MS symptomatology (e.g. constipation) rather than affecting overall disease progression directly; and mental health, since depression has been identified as a risk factor for RRMS disability and relapses.^78,79,80^

## C. Commensal therapy

### Major trends

The major findings for each of the commensal therapy animal studies included in this review can be found in Table 4. A qualitative summary of these studies is provided below, followed by a semi-quantiative ranked evaluation using BH criteria.

No human studies were identified for commensal therapy in this review, so the below trends are limited to preclinical findings. All but two (*L. reuteri^37^*^,38^ and *Allobaculum^38^*) of the commensal organisms studied among the nine studies were shown to delay EAE onset and decrease clinical scores, incidence, inflammatory CNS infiltration, and demyelination. *P. histicola* was one of two commensals represented in more than one study and exhibited positive outcomes in each.^56–58^ One study also reported reduced astrocytosis and microglial activation in the brain and spinal cord of *P. histicola*-treated mice,^58^ while a sister study found that *P. histicola* helped to strengthen the MGBA by decreasing BBB permeability and restoring gut permeability.^56^ Similar to probiotic therapy, reduced proinflammatory and increased anti-inflammatory immune responses were observed in each of the commensal studies. Specifically, studies found decreased IL-17- and IFN-γ-producing CD4 + T cells,^56,58,65^ T_H_17 cells,^65^ and IL-17,^56,62,70^ IFN-γ,^56,70^ IL-23,^56^ and IL-12^70^ cytokines; and increased IL-10,^56,62,70^ TGF-β,^56^ and Tregs.^56–58,65^ These results were consistent across all three *P. histicola* treatments.^56,57,58^ *L. reuteri* was also represented in multiple studies and was shown to exacerbate EAE in both, either when administered alone (in the context of a normal microbiome)^37^ or in combination with *Allobaculum* (in a dual-colonization gnotobiotic model).^38^

For studies that analyzed the microbiome, commensal therapy groups had a general microbiome shift toward pre-EAE states following treatment, including an increased relative abundance of *Bacteriodetes, Firmicutes, Prevotella* spp., and *Lactobacillus* spp.^56,57,65^ Mechanistically, the adoptive transfer of splenocytes from *P. histicola*-treated mice led to decreased EAE incidence in recipient mice.^56^ Similarly, the adoptive transfer of FoxP3+ cells from wild-type *B. fragilis*-treated mice, resulted in decreased EAE clinical scores and increased levels of IL-10 in recipient mice.^62^ These findings were not observed in the recipient mice receiving cells from polysaccharide A (PSA)-deficient *B. fragilis*-treated mice, suggesting that PSA is requisite for EAE protection. Separately, treatment with *C. butyricum* was reported to suppress phosphorylation of p38 MAPK and JNK signaling pathways – which are typically elevated in EAE – in the spinal cords of mice.^65^ One commensal was also found to be at least as effective as two different DMTs (glatiramer acetate^57^ and IFNβ^58^).

### BHC scores and rankings

The BH score calculations and findings for commensal therapy are detailed in Supplemental File 2, and summarized in Table 5.

Treatment with *P. histicola* had fairly strong evidence (BH score = 7), but fell short across several BH categories (BHC #s 4 and 6), as it lacked human studies and replication by independent groups (Table 5). Another promising treatment with a high BH score was *B. fragilis* (BH score = 5), which scored points in BHC # 1, 2, 5, 7, and 8 owing to an adoptive transfer experiment, but lacked replication and evidence of dose-response and cessation effects. The remaining treatments were characterized by low BH scores (ranging 1–4) comprised of points in BHC # 1, 2, 3, and/or 5, once again resulting from a paucity of studies, lack of mechanistic evidence, and/or presence of conflicting evidence, as observed with *L. reuteri* (BH score = 1), which was found to exacerbate the disease in three of the five studies, leading to a 2-point deduction for BHC #4. As noted with *L. casei* for probiotic therapy, this interpretation is confounded by the use of different strains/isolates and modes of treatment (stable commensal colonization vs. daily gavage) across studies and would benefit from careful standardization (Table 5).

### BHC deficiencies

Using the Bradford Hill criteria, commensal therapy had strong evidence for temporal relationship, specificity of association, and biological plausibility, but was lacking in the remaining categories (BHC #s 2–4, 6, and 8; Table 6). Additional studies replicating current findings and testing more commensal organisms and combinations should be the main focus of future studies, since there were few commensal therapy studies overall and only *P. histicola* and *L. reuteri* were used in more than one study. Future studies should also focus on confirming colonization of the commensal organisms in the gut to reduce confounding and strengthen the specificity of association (BHC #7). Testing the effects of live versus heat-killed organisms and their products should also be prioritized, as these findings may contribute to the elucidating the underlying therapeutic mechanisms. For instance, subsequent studies of *B. fragilis* by the same research group utilized only the *B. fragilis* PSA symbiosis factor rather than administering live, wild-type *B. fragilis* and found similar reductions in EAE severity, as well as protection against EAE demyelination and inflammatory responses, providing a key molecular mechanism in support of the action of the live bacterium.^81–84^

Other recommendations reflect those of probiotic therapy, namely controlling for alternative explanations, supporting immunological and microbiological findings with mechanistic experiments, and adding standardization to promote study design consistency and ease of comparison across studies.

## Discussion

The purpose of this comprehensive review was to compile, summarize, and systematically rank the current evidence for probiotic and commensal therapeutic efficacy in MS and its preclinical models in an effort to identify weaker areas that should be addressed in future studies. A total of 37 studies were evaluated, including 28 for probiotic therapy and 9 for commensal therapy. The probiotic formulations VSL#3 (BH score = 9), *B. animalis, L. paracasei*, and *E. coli* Nissle 1917 (BH scores = 7) ranked highest due to their fulfillment of at least six of the eight Bradford Hill criteria. For commensal therapy – which suffered from a complete absence of clinical studies – the highest rankings went to *P. histicola* (BH score = 7) and *B. fragilis* (BH score = 5).

Animal studies demonstrated generally higher efficacy for reducing disease severity and progression with probiotic therapy than did the human studies, which is not unexpected, given the known shortcomings of the animal models, and the expected difficulties in translating basic science findings into therapy. The disconnect between human and animal studies could also be due to the difference and extent of the clinical markers measured, as clinical studies only measured MS severity through EDSS and questionnaires, while the pre-clinical studies were able to investigate EAE and the other MS models more comprehensively. MRI evaluation is a powerful, unbiased, and quantitative surrogate for MS severity and progression, and this was conspicuously lacking in the human studies. Additionally, human studies were in all likelihood underpowered to detect potentially subtle effects of probiotic treatments, and confounded by multiple environmental variables (e.g. diet, host baseline differences) that are impossible to control in this setting.

Replication of findings (BHC #4) was one of the most deficient Bradford Hill criteria across studies, with only 25% (*n* = 28) of the formulations receiving points and two of them (*L. reuteri* and *L. casei)* losing points. Another almost uniformly unfulfilled criterion was cessation of exposure, only addressed by six formulations. Both probiotic and commensal therapies would benefit from additional replication, testing more organism combinations, improved mechanistic evidence and comparison of live versus heat-killed organisms and their soluble products, and protocol standardization to enable improved contextual comparison across studies.

### Limitations

#### Study limitations

There were several limitations to accurately assessing the efficacies of both therapies. First, there was widespread study design variability in the species and/or strain, dosage, duration of intervention, timeline, and sample characteristics. These variations would have been beneficial to external validation if the same strains were used across studies, but instead posed a challenge for assessing therapeutic utility. Standardized protocols outlining the optimal dosage, timeline, and duration for different organisms would be helpful for mitigating this issue, as was the focus of a review on probiotic therapy that concluded 10^9^ CFU for 8–12 weeks duration produced the most favorable results.^31^

Another study design issue was exclusion criteria and control of confounding variables, since some of the human studies did not account for diet or stress, which can alter gut microbial composition and subsequently influence MGBA interactions and concurrent DMT use, which could overshadow the true therapeutic efficacy if synergism exists between the two.^34^ Additionally, genetic variability was also mostly unaccounted for in both human and animal studies (since the latter for the most part used a single strain of mouse). Furthermore, none of the human studies were conducted long enough to span the average remission period of 12–18 months, so the true impact of each therapy on reducing the severity of MS cannot be revealed with certainty.^1,85^ As for animal studies, none of these can accurately capture the complexity of spontaneous MS and its various forms in humans.^86–88^

Only five studies tested the effects of live versus heat-killed organisms or their products.^48,49,53,56,62^ This distinction is important, since equivalent efficacy with heat-killed organisms would help to reduce any associated risks of therapy posed by live microbiota and likely improve treatment uptake and adherence in patients. Furthermore, this effect likely differs across organisms. For instance, *L. paracasei*,^49^ a combination of *L. crispatus* and *L. rhamnosus*,^53^ and a combination of two *B. animalis* subsp. lactis strains^53^ did not need to be viable for EAE suppression, while *P. histicola*^56^ and Lacto-mix^48^ did. Additionally, protection from EAE elicited by *B. fragilis* required the expression PSA by this bacterium, indicating that this bacterial product alone can play an important role in EAE protection.^62^ Indeed, follow-up studies confirmed that live *B. fragilis* is not required, while PSA is sufficient to elicit a therapeutic effects.^81–84^ Future studies should prioritize these distinctions to help optimize efficacy and therapeutic success.

Another limitation of this review was the quality and risk of bias for the studies included. Most of the animal studies included were classified as “medium quality” with “uncertain” bias due to the lack of explicitly stated randomization and blinding measures used. A lack of randomization can subject the results to inadvertent confounding and chance findings.^39,40^ Animals that live together in the same cage or area of a room may have more similar characteristics to each other than compared to a different cage or area importantly including the basal composition of their gut microbiomes. Additionally, a lack of blinding can introduce both performance and detection bias, as a researcher or caretaker’s knowledge of the treatment group can cause them to subconsciously act differently toward one group, such as providing extra care to sicker animals.^39,40^ It is entirely possible that these studies did in fact incorporate these measures into their protocol, but since it was not stated it was considered to be absent for this review. Other issues regarding quality include the premise that the positive findings observed across studies may simply reflect consistency of confounding variables and/or publication bias, rather than the therapy itself.

#### Review limitations

As for the risk of bias for this review, there were several methodological flaws in the assessment of therapeutic efficacy. Personal judgment was required for assessing each therapy’s fulfillment of the Bradford Hill criteria and there were no pre-defined guidelines as to what should be considered “sufficient evidence.” The scoring system was implemented as an arbitrary method to facilitate comparison of the efficacies in the context of the recommendation criterion and not intended to be a comprehensive assessment of the therapies. Regardless, the aim of this review was to highlight areas within each therapy that should be strengthened in future studies, so the risk of bias in this sense seems low. Separately, a few of the Bradford Hill criteria may be less important for establishing efficacy, causing the therapeutic to be penalized for lacking evidence in a non-applicable category. For example, a threshold effect rather than a dose–response relationship might be necessary for observing beneficial effects. Cessation of exposure may also not be necessary to demonstrate, since these therapies would theoretically be lifelong as is the case for DMTs. Accordingly, another avenue for future research could be establishing a minimum set of probiotic specific criteria for comprehensively evaluating therapeutic strategies in both animal and human studies.

Other limitations of this review were related to the search and screening process for identifying relevant studies. First, the use of an English language filter in our search strategy imposed obvious restrictions on the number of studies included and extent of available evidence. Second, the operational definitions we established limited the scope of probiotic and commensal therapies to only live or heat-killed bacteria, excluding any mechanistic evidence that may have been generated in studies that used only probiotic/commensal strain-soluble products. The BH score for *B fragilis*, for example, could have been improved had the follow-up studies that focused on the *B. fragilis* PSA symbiosis factor been eligible for inclusion.^81–84^ Lastly, we did not contact the authors of studies classified as medium or low quality for clarification of missing methodological data (i.e. randomization, blinding). Attaining such information could have altered our quality assessments and evaluations for BHC #2. Regardless of these limitations, this review was intended as a resource to guide and optimize future probiotic and commensal therapy studies by highlighting both emerging therapies and study shortcomings, rather than to firmly conclude the therapeutic utility of specific formulations.

## Conclusion

In this comprehensive review, we used a Bradford Hill criteria scoring approach to provide a multi-parameter assessment and ranking of evidence for specific gut microbial therapies, with the overall goal of identifying and highlighting areas of need for future research (see Tables 5 and 6). Several formulations emerged as having the most promise, including VSL#3, *B. animalis, L. paracasei*, and *E. coli* Nissle 1917 for probiotics; and *P. histicola* and *B. fragilis* for commensals. However, many other therapies fell short across a number of criteria, notably replication of findings. Other Bradford Hill criteria lacking evidence were temporal relationship and specificity of association for probiotic therapy, and strength of relationship, dose–response relationship, and coherence for commensal therapy. Future studies should prioritize addressing these shortcomings through better control of confounding, supporting immunological and microbiological findings with mechanistic experiments, improved standardization of protocols and therapeutic formutions, and the other suggestions discussed in this review. Focusing on these areas is necessary to make progress toward clinical implementation, since cheaper, safer, and more durable treatments for MS are in demand.

## Supplementary Material

Supplemental Material
