# Supplementary material for: Probiotic and commensal gut microbial therapies in multiple sclerosis and its animal models: a comprehensive review
Source: Gut Microbes. 2021 Jul 15;13(1):1943289. doi: 10.1080/19490976.2021.1943289 (PMC8284149; doi:10.1080/19490976.2021.1943289)
Supplement: Supplemental Material [file KGMI_A_1943289_SM8746.zip › Supplementary information/Supplementary caption.docx]

**Supplementary File** **1**

A. **Search Strategies** – Search terms, Boolean operators, and limits used for each of the four databases.

B. **PRISMA Table** – Tracks the identification, screening, eligibility, and inclusion of studies from first, second, and combined search results of all four databases.

C. **Compiled Human Studies** – List of all human studies assessed for eligibility and raw data extracted from those included.

D. **Cochrane ROB Tool** – Quality assessment results of human studies using the Cochrane ROB tool.

E. **Compiled Animal Studies** – List of all animal studies assessed for eligibility and raw data extracted from those included.

F. **SYRCLE Tool** – Quality assessment results of animal studies using the SYRCLE tool.

G. **Study Categorization** – List of included studies categorized into their respective pipelines: probioitic vs commensal, human vs animal, low vs. medium vs high quality, and therapeutic vs prophylactic vs both.

H. **Study Characteristics** – Analysis of study metrics, including study design and location, bacterial species/strains and dosages, animal models, human participant characteristics, quality metrics, intervention duration, measurements, and statistical analyses.

I. **Positive vs Negative & Neutral** – Categorization of study outcomes as “positive” or “negative/neutral” based on the reported effects of each intervention.

J. **Supporting & Discrepant** – Studies were grouped by bacterial formulation to highlight similarities and discrepancies in study design and major findings.

K. **Major Trends** – Major trends for clinical, immunological, microbiological, and mechanistic outcomes were summarized across the human vs animal pipeline for probiotic and commensal therapies.

**Supplementary File 2 — BH Evaluation** – Assessment of overall quality and strength of evidence using the Bradford Hill criteria, including temporal relationship, strength of relationship, dose-response relationship, replication of findings, biological plausibility, cessation of exposure, specificity of association, and coherence between multiple approaches.
